# Activated B-Cells enhance epitope spreading to support successful cancer immunotherapy

**DOI:** 10.3389/fimmu.2024.1382236

**Published:** 2024-03-19

**Authors:** Guillaume Kellermann, Nicolas Leulliot, Julien Cherfils-Vicini, Magali Blaud, Patrick Brest

**Affiliations:** ^1^ Telomium, SAS, Ivry-sur-Seine, France; ^2^ Université Paris Cité, Centre national de la recherche scientifique (CNRS), Cibles Thérapeutiques et Conception de Médicaments (CiTCoM), Paris, France; ^3^ Université Côte d’Azur, Institute for Research on Cancer and Aging, Nice (IRCAN), Centre national de la recherche scientifique (CNRS), Institut national de la santé et de la recherche médicale (INSERM), Centre Antoine Lacassagne, Institut Hospitalo-Universitaire (IHU), RESPIRera, Fédérations Hospitalo-Universitaires (FHU)OncoAge, Nice, France

**Keywords:** B-cell, tolerance, immunotherapy, epitope spreading, RNA binding protein

## Abstract

Immune checkpoint therapies (ICT) have transformed the treatment of cancer over the past decade. However, many patients do not respond or suffer relapses. Successful immunotherapy requires epitope spreading, but the slow or inefficient induction of functional antitumoral immunity delays the benefit to patients or causes resistances. Therefore, understanding the key mechanisms that support epitope spreading is essential to improve immunotherapy. In this review, we highlight the major role played by B-cells in breaking immune tolerance by epitope spreading. Activated B-cells are key Antigen-Presenting Cells (APC) that diversify the T-cell response against self-antigens, such as ribonucleoproteins, in autoimmunity but also during successful cancer immunotherapy. This has important implications for the design of future cancer vaccines.

## 1 Introduction

The breakthrough in cancer immunotherapy was awarded by the Nobel Prize in Physiology or Medicine to James Allison and Tasuku Honjo in 2018 (1). Immune checkpoints Therapies (ICT) have fundamentally changed cancer treatment approaches over the past decade. ICT have become a robust method to reinvigorate exhausted T-cells targeting neoepitopes on cancer cells. Neoepitopes occurrence in tumors correlates with tumor mutation burden (TMB). Some neoepitopes are recognized as foreign antigens by T-cells and become major drivers of antitumoral immunity when their activity is prolonged by ICT [1].

However, the delay between treatment initiation and response is a weakness. Indeed, the benefit of ICT is not apparent until 2-3 months, and sometimes not until 6-12 months after treatment initiation, comparing unfavorably to chemotherapy (2, 3). Therefore, ICT is often less effective for very aggressive tumors. Combining ICT with intra-tumoral injections of the replicating virus T-VEC (FDA-approved), failed to accelerate therapeutic responses: the mean time to response was 8.4 months (4). In a HNSCC trial combining T-VEC and ICT, 27.8% of patients died before the first evaluation at week 9 (5), highlighting the need to accelerate the induction of functional antitumor immune responses. Moreover, the majority of patients do not respond at all to ICT, because most tumors do not present enough neoepitopes. Persistent clonal neoepitopes, required for long term remissions, are often missing (6, 7), and inefficient epitope spreading to non-mutated tumor associated-antigens (TAA), explains the primary, but also secondary resistance to ICT (8).

Developing diversified immune responses is critical to overcome the limitations of current immunotherapies. Understanding why and how epitope spreading occurs holds the key. In this review, we take an evolutionary approach to provide a unifying explanation for this phenomenon and describe the key pathways driving it. By leveraging these insights, future immunotherapies can be designed to activate these pathways and unleash the full potential of the immune system against cancer.

### 1.1 Break of tolerance during immunotherapy

Neoepitopes and chronic inflammation in some tumors break immune tolerance to tissue-specific, non-mutated, self-antigens (8), leading to serious adverse events when these tumor antigens are expressed in other normal tissues (9). Nevertheless, these autoimmune side effects often correlate with survival in patients treated with ICT (10). This suggests that these reactions are not only adverse events but also real contributors to the therapeutic effect (11). They however remain difficult to predict. Self-tolerance does not impede the generation of activated T-cells against many self-antigens, but may strongly impair their functional activity in tissues (12). Consequently, spontaneous immune responses occurring naturally against self-associated tumor antigens, or T-cell responses elicited by cancer vaccines do not consistently correlate with therapeutic outcomes observed *in vivo* and can’t be used to prove or rule out a break of immune tolerance, not even for neoepitopes (13).

Rather than relying on unpredictable, rare, delayed, poorly targeted, and potentially dangerous autoimmune reactions, a more rational immunotherapy should directly break immune tolerance to specific cancer targets. This would allow safer, faster, and more reliable tumor regression regardless of TMB or neoepitope load. This was the original goal of cancer vaccines, but their inability to reliably break immune tolerance, in clinical conditions, has so far limited their efficacy, except in the rare patients experiencing epitope spreading (14).

**Figure 1:**
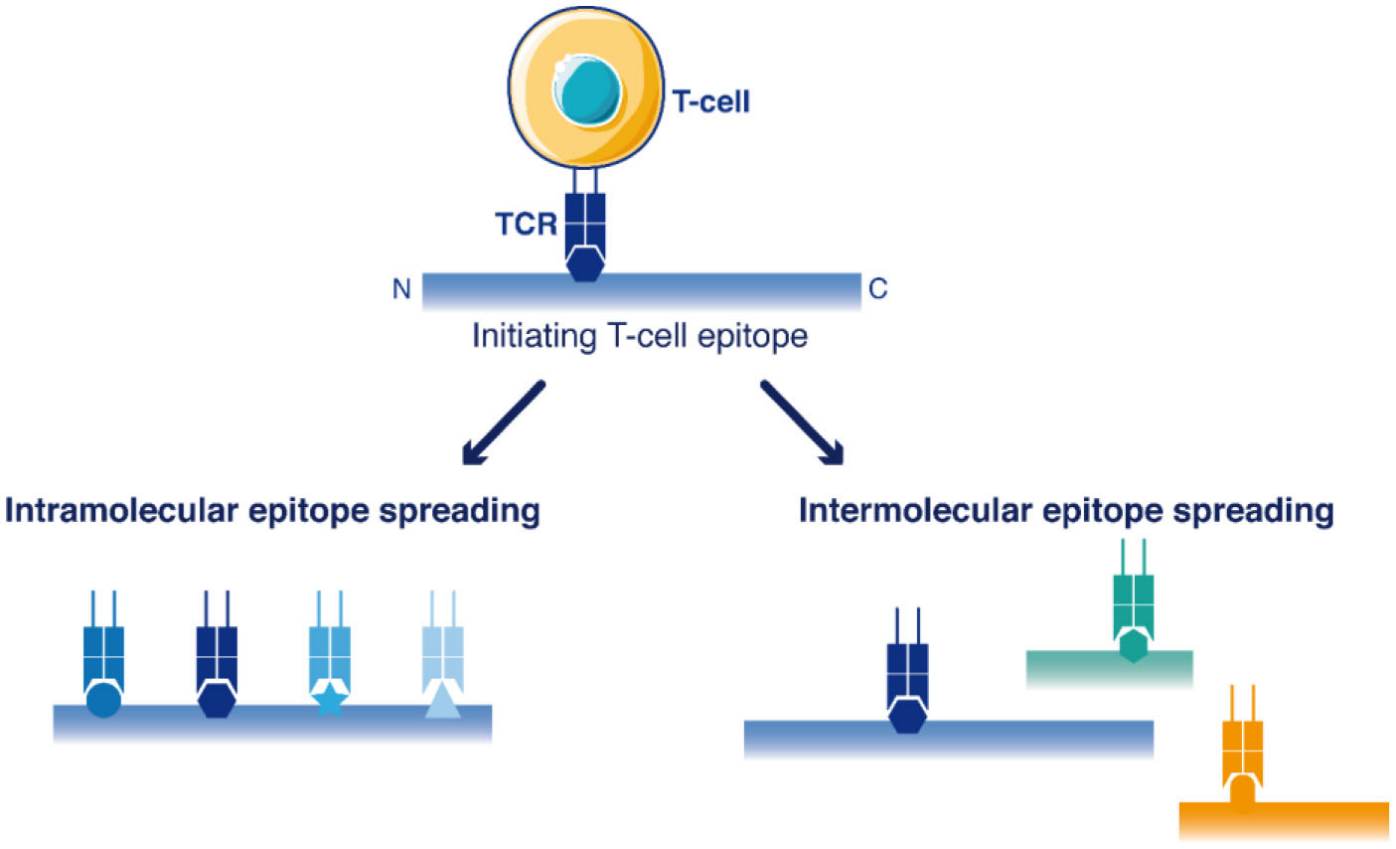
Schematic view of T-cell epitope spreading into the sequences of antigens. The primary immune response targets dominant initiating T-cell epitope(s). Then, the response may be further extended to other T-cell epitopes within the same antigen (intramolecular epitope spreading) or among other antigens (antigen spreading/intermolecular epitope spreading).

Epitope spreading is a diversification of the initial T-cell response against novel epitopes that are different from, and not cross-reactive with the original epitope(s) targeted in the primary immunization (**Figure 1**). Epitope spreading can occur to cryptic epitopes of the same targeted antigen (intra-molecular spreading) or against other antigens (inter-molecular spreading or antigen spreading) (15). Antigen spreading against other immunodominant epitopes readily occurs through bystander activation during many immune responses, but this is usually unrelated to a break of immune tolerance. On the contrary, intra-molecular diversification of the T-cell response reported for self-antigens such as MAGE Family Member A3 (MAGEA3) (16) or Telomerase (TERT) (17) provided stronger evidence that a true break of immune tolerance occurred in the few patients successfully reacting to cancer vaccines. Epitope spreading occurred in these patients several months after the initial vaccination, and this latter diversification of the T-cell response was correlated with the appearance of objective therapeutic responses, as if the vaccine-induced T-cells were not the direct mediators of tumor regression, but rather initiators of a broader T-cell response after several cycles of epitope spreading (16–18). It is therefore crucial to identify which cell populations are promoting such epitope diversification.

### 1.2 Activated B-Cells promote epitope spreading

As early as 1993, Charles Janeway hypothesized that B-cells are the key antigen-presenting cells (APC) behind the diversification of T-cell responses (19). In the last three decades, many studies have supported the key involvement of B-cells as critical APC for T-cell activation in many autoimmune diseases (20–25). The fundamental role of auto-reactive B-cells as a key antigen presenting cell (APC) to activate T-cells is widely established in autoimmune diseases (20–24), where B-cells have even become a therapeutic target (24, 26). B-cells diversify CD4 T-cell responses (22), cross-present antigens during autoimmunity (27, 28) and also help CD8 T-cell independently of antigen presentation (29). However, in cancer immunotherapy, epitope spreading has been mostly attributed to dendritic cells (DC) (30). The anti-tumoral role of B-cells has long been neglected, because tumor mouse models have mainly predicted a pro-tumoral role of B-cells, in contrast to the situation in humans (31, 32). However, recent data has strengthened the evidence for a strong correlation between B-cell activation and successful immunotherapy in humans (33–38). This suggests that B-cells play a key role in tumor antigen presentation (39) and are critical for successful cancer immunotherapy (32, 40) and also to the associated-autoimmune reactions (11, 25).

### 1.3 The viral escape theory: a potential fundamental reason for epitope spreading

During a primary viral infection, DC capture and present foreign antigens to activate high-avidity T-cells that kill infected cells. Later, serum antibodies rise to suppress viral dissemination and prevent recurrence. However, when the virus escapes through random mutations at protective T-cell epitopes, B-cells still capture virus particles released from infected cells resistant to T-cell killing (41). In this situation, B-cells are continuously activated via their BCR (B-Cell Receptor) and present antigens to T-cells. Thus, persistent antigen presentation by memory B-cells means that an immune response has been induced, but also that this immune response has failed to completely remove the pathogen. Therefore, these conditions may have been evolutionarily selected to provide special signaling to recruit a new wave of effectors, that are more likely to cause autoimmune damage, but are able to remove escape variants that cannot be cured with the primary response (42–45).

High-avidity T-cells reacting against self-antigens are deleted by central tolerance, but low avidity-cells T-cells are not deleted. They are held in reserve in the case the primary response fails (42, 46–48). With the help of B-cells, T-cells with gradually lower TCR affinity are progressively recruited (45), which may ultimately break immune tolerance and cause autoimmune damage. During stepwise expansion of the immune response, the hierarchical order of epitope spreading starts with the most immunodominant epitope and then extends to the least dominant (15). Activated T-cell reaching subdominant epitopes and cryptic epitopes that have not been tolerized break immune tolerance (49, 50).

B-cells are highly efficient at reconcentrating diluted antigens released from dead cells by capture through their BCR which allows the selective presentation of a specific antigen to activate T-cells (19). On the contrary, DCs capture and present antigens non-specifically. Therefore, DCs need to remain under the strict control of T regulatory cells (Treg) (51), whereas B-cells are less stringently controlled by Treg (52, 53). Indeed, B-cells have a unique ability to activate T-cells and support the differentiation of low-avidity T-cells (44, 45) which are normally suppressed by T-regulatory cells (Treg) (54, 55). DC play a crucial role in the initial T-cell activation, but B-cells, not DC, uniquely support CD4 T-cells cooperation and their further activation (22, 56) through the OX40-OX40L pathway (22, 57). Memory B-cells act as specific APC that overcome the suppressive activity of Treg in many autoimmune disorders (23, 58, 59).

Activated B-cells also provide additional APC numbers to bypass T-cell competition, reduce immunodominance and diversify the T-cell response. Indeed, during viral infections, T-cells compete for APC and costimulatory molecules, establishing a state where only a few epitopes dominate the T-cell response. Dominant epitopes usually display a high affinity for HLA and provide the strongest T-cell activation (60), but immunodominance can be bypassed by artificially increasing the number of APC (61–63). During an infection, if the antigen is only presented by dendritic cells (DCs), the number of activated APC is limiting, therefore creating a competition between T-cells for the few activated APCs and favoring the dominance of a T-cell response against only a few epitopes. On the contrary, expanded B-cells can outnumber DC and provide an increased number of APC that relief the competition for APC. This bypasses the immunodominance and support the extreme diversification of the immune response to multiple new epitopes: epitope spreading (45). Moreover, plasma B cells secrete high levels of antibodies that bind the antigen and facilitate the subsequent mobilization of other non-conventional APC (64), further enhancing antigen presentation and cross-presentation which favors the break of tolerance (65).

Broad immune responses targeting multiple epitopes are more efficient than restricted responses, but carry a higher risk of autoimmunity (66). While the risk-benefit ratio of inducing a broader T-cell activation may not always be positive during a chronic infection, a live pathogen that acutely escape immune control and becomes detected as a threat out-of-control, provides a stronger signal than a chronic infection (67). Acute reinfection with a pathogen expressing a self-antigen, but not a standard DC vaccine, can directly activate low avidity T-cells and induce autoimmunity after only one week (47), demonstrating that epitope spreading is not necessarily a slow process but can be accelerated. However, this also suggests that standard vaccine technologies and current adjuvants are suboptimal for inducing epitope spreading. It is therefore crucial to identify the most suitable signaling pathways that, can efficiently drive epitope spreading.

### 1.4 The unique role of TLR7 in B-cells to break tolerance

Toll-like receptor (TLR) -9 or TLR3 agonists (CpG and PolyI:C) are usually scored as the more powerful adjuvants for standard antigens in mice, but a TLR7 agonist was reported as the most effective adjuvant for a self-antigen (68). TLR7, a sensor for viral RNA, has a superior ability to induce IL-6 dependent resistance to the suppressive activity of Treg (69, 70). TLR7 agonists are more effective in bypassing Treg than agonists for TLR4, TLR5 (71) and even TLR3, RIG-I, MDA-5 (68, 72).

TLR7 plays an essential role in B-cells unlike any other TLR, including TLR9, despite both receptors rely on MyD88 signaling (73). TLR7, but not TLR2, TLR3, TLR4 or TLR9 is required for the formation of spontaneous germinal centers *in vivo* (74). TLR7 agonists, but not TLR3, TLR4 or TLR9 agonists, induce the accumulation of atypical memory CD11c+ B cells (ABCs), associated with autoimmunity (75).

The major importance of TLR7 in the break of immune tolerance is strengthened by the independent discovery that several genetic/epigenetic variations in TLR7 leads to autoimmunity (76–78). Indeed, at the genome level, a single gene duplication in TLR7 induces autoimmune pathology, demonstrating that TLR7 levels must remain tightly regulated *in vivo* (79). At the transcription level, type II interferon potentiates the TLR7 pathway (80, 81) and type I interferon up-regulates TLR7 expression 40-fold in B-cells, without changing the levels of other TLR, thereby increasing the sensitivity of B-cells specifically against RNA viruses (82). Of note, RNA viruses have a higher mutation rate than any other pathogen. They pose the risk of escaping the immune response by random mutation (41). As a result, TLR7 signaling in B-cells seems to have been evolutionarily selected to respond efficiently against viral escape variants by facilitating epitope spreading.

### 1.5 RNP: optimal inducer of the dual BCR/TLR7 signaling

Ribonucleoproteins (RNPs) are RNA-protein complexes that can be mistaken as RNA virus. They constitute a major class of self-antigens targeted in autoimmune diseases (83). Autoantibodies against different RNPs antigens are frequently found in various autoimmune diseases. However, mice genetically deficient in secreting antibody showed that serum antibodies are not essential to the autoimmune pathology. Rather, it was found that the presence of non-secreted auto-antibodies at the surface of B-cells (the BCR) is critical, suggesting that it is the APC function of B-cells which is key for autoimmunity (21, 22, 84). Genome-wide association studies have also identified the BCR signaling among the most frequent genetic polymorphisms predisposing to autoimmunity (20).

**Figure 2:**
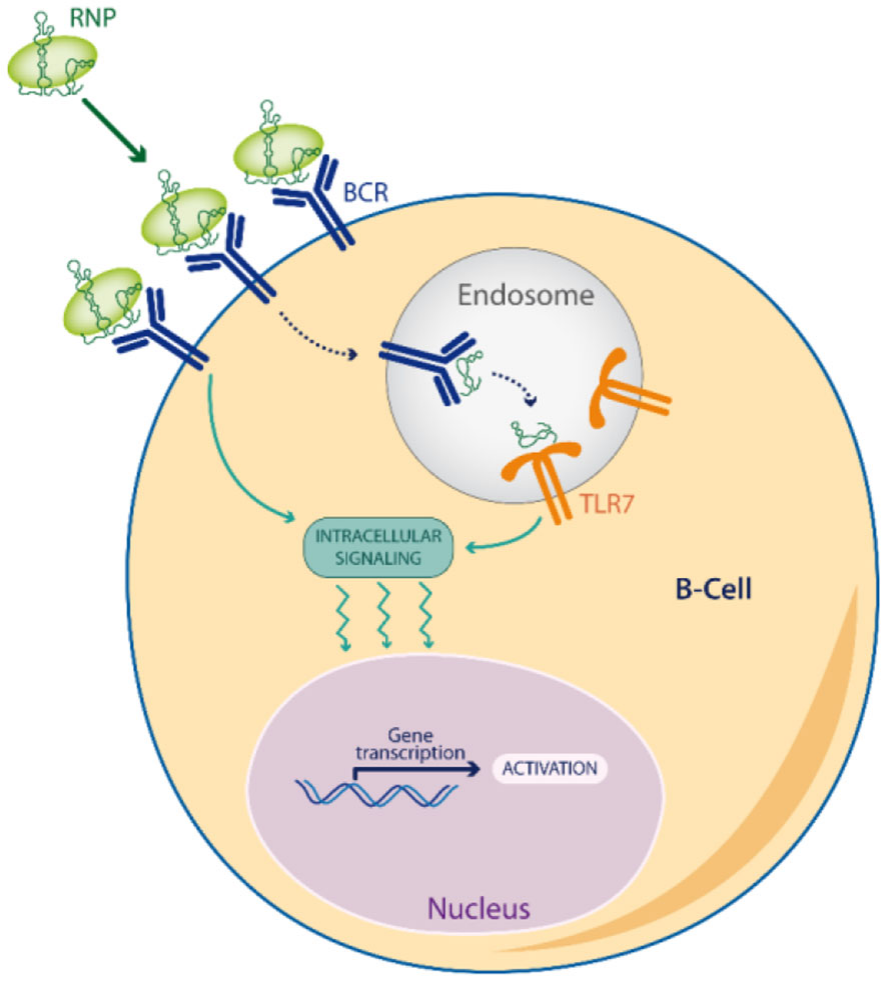
Dual BCR/TLR signaling. Viral Escape Mimetics (VEM), like RNPs antigens, induce a dual BCR/TLR signaling in memory B-cells which enhances antigen presentation to T-cells, and favors a break of tolerance by epitope spreading.

Noticeably, RNPs induce a dual BCR/TLR signaling (**Figure 2**), by the sequential engagement of the antigen signaling at the cell-surface (BCR or FcR through their protein part), followed then by TLR7 signaling in endosomes (through their RNA part) (78). This acute or prolonged dual signaling by RNPs better mimics a RNA virus escaping immune control than any other self-antigen, explaining why RNP often break tolerance by epitope spreading (83).

The co-stimulation of the BCR is required to unleash the full potential of TLR7 signaling. Otherwise, TLR7 activation alone is rapidly restrained to hypo-responsiveness (85). Antigens that activate the dual BCR/TLR7 signaling, but not dual BCR/TLR9 or TLR7 alone, promote B-cell differentiation into plasma cells (86), found to correlate with antitumor responses to anti-PD-L1 (37). Noticeably, the dual BCR/TLR7 signaling has also been experimentally found as the optimal combination for vaccination with human B-cells (87). Current studies support a model where the dual activation of the BCR/TLR7 signaling by a foreign RNP antigen in B-cells is the first event that orchestrates the break of tolerance in several autoimmune diseases (83, 88), suggesting a cooperation between the three main autoimmune mechanisms: molecular mimicry, bystander help and epitope spreading.

Ribonucleoprotein antigens are also the major class of self-antigens targeted by B-cells in tumors (89). Among the most studied TAA, telomerase is a RNP absent in most normal adult tissues but reactivated in most cancers and essential for prolonged tumor growth and metastasis. After vaccination with a single peptide from TERT, extensive epitope spreading into the entire TERT protein has been observed in the few patients achieving tumor regression, but not in non-responders (17). In these responders, the T-cell response against TERT was so widespread that T-cell reactivity was detected at almost half of the positions tested along the protein, providing evidence of profound epitope spreading (17). Interestingly, the long-term survival of these patients without autoimmune extension to other tissues (90), illustrates the concept of beneficial autoimmunity (11). This supports the development of immune activators capable of directly eliciting such extensive and diversified immune responses in the majority of patients.

## 2 Conclusions and perspectives

No cancer vaccine has been specifically designed to break immune tolerance against a tumor antigen by inducing intramolecular epitope spreading through the specific activation of the dual BCR/TLR7 signaling in B-cells. All mRNA/DNA/viral cancer vaccines suffer from a de-coupling of timing and localization between early TLR/IFN stimulation in endosomes and the delayed antigen expression in the cytoplasm (91). Current cancer vaccines do not sequentially activate the BCR at the cell-surface, followed by connected TLR7 activation and MHC antigen presentation in the resulting endosome (28). Neither unassociated adjuvants commonly co-administrated in protein/peptide vaccine formulations, nor antigens delivered by mRNA/DNA/viral vectors appropriately reproduce the temporospatial trafficking of a viral particle captured by an APC.

Vaccines based on antigen-adjuvant conjugates should provide better Viral Escape Mimetics (VEM) to induce epitope spreading. Recently, peptides linked to the synthetic TLR7 agonists (imidazoquinoline) have been tested (92). However, small peptides do not contain enough B and T epitopes linked on the same antigen to support direct epitope spreading, while the *in vivo* adjuvancy of imidazoquinolines is vastly inferior to RNAs, because of abnormal TLR7 trafficking when TLR7 is activated by these small synthetic agonists instead of an RNA (93). Larger protein-RNA complexes should be preferred, as antigens close to a viral size display an optimal trafficking into lymph nodes, contrary to small antigens (94). Finally, many of the current cancer vaccines still use oil adjuvants which induce antibody production by B-cells that prevent infectious diseases, but abrogate the antitumoral activity of B-cells (95) and the response to ICTs (96). Therefore, future cancer vaccine must activate the most potent type of B-cells for ICT.

Vaccines against neoepitopes can induce broader immune response, but they have nevertheless mostly failed to solve the primary resistance to ICTs (97), possibly because a standard immunization approach doesn’t reverse self-tolerance (12, 47). Many neoepitopes do not display enough foreignness and several work as suppressive regulatory T cell epitopes (98). Therefore, in most patients, ICTs can only become efficient with a break of immune tolerance induced by profound epitope spreading against native non-mutated epitopes, a process lead by activated B-cells, as evidence in autoimmunity.

## Author contributions

GK: Funding acquisition, Visualization, Writing – original draft, Writing – review & editing. NL: Funding acquisition, Visualization, Writing – original draft, Writing – review & editing. JC-V: Writing – original draft, Writing – review & editing. MB: Funding acquisition, Visualization, Writing – original draft, Writing – review & editing. PB: Funding acquisition, Visualization, Writing – original draft, Writing – review & editing.

## Glossary

**Table:**

ABCs	atypical memory B cells. They are also called extrafollicular, inflammatory double negative, or age-associated B cells. They usually express CD11c, Tbet, FcRL4 but lack CD21, CD27 and IgD.
APCs	Antigen-Presenting Cells (ex: dendritic cells or B-cells).
BCR	B-Cell Receptor.
Cancer immunotherapy	treatments that use the host immune system to fight cancer.
DC	Dendritic cells.
ICTs	Immune checkpoints Therapy are monoclonal antibodies targeting critical regulators of the immune system like PD-1/PDL-1, CTLA4, LAG-3 that revigorate exhausted T-cells and prolong their activity. Some cancer cells also exploit this regulation to protect themselves, but this cancer-associated inhibition may be canceled with ICTs.
RNP	Ribonucleoprotein (protein-RNA complexes).
T-cell exhaustion	T cells often met during chronic pathology, where T-cells display poor effector function to prevent immunopathology.
Neoepitopes	mutated epitope.
TAA	tumor associated-antigens.
TERT	Telomerase Reverse Transcriptase.
Treg	T-regulartory cell.
TLR	Toll-like receptor.
VEM	Viral Escape Mimetics are antigens that sequentially induce the dual BCR/TLR signaling in memory B-cells. Acute or persistent dual BCR/TLR signaling means that preexisting immune responses already exist against this antigen, but has failed to remove it, which provides a specific new signaling to induce a more profound immune response: epitope spreading.
